# Supplementary material for: Prevalence of infantile wheezing and eczema in a metropolitan city in Japan: A complete census survey
Source: PLoS One. 2022 May 5;17(5):e0268092. doi: 10.1371/journal.pone.0268092 (PMC9070868; doi:10.1371/journal.pone.0268092)
Supplement: Attachment — Submitted filename: Response_to_Reviewer.pdf [file pone.0268092.s001.pdf]

FOR JOURNAL REQUIREMENTS

1. Please ensure that your manuscript meets PLOS ONE's style requirements, including those for file naming.

RESPONSE: We have made changes to our manuscript according to the *PLOS One* format.

2. Please provide additional details regarding participant consent. In the ethics statement in the Methods and online submission information, please ensure that you have specified (1) whether consent was informed and (2) what type you obtained (for instance, written or verbal, and if verbal, how it was documented and witnessed). If your study included minors, state whether you obtained consent from parents or guardians. If the need for consent was waived by the ethics committee, please include this information.

RESPONSE: We have added the following sentences about participants' consent: (Line 124) "Parents of participants provided written consent on the questionnaire for using the data for epidemiological studies."

3. In your Data Availability statement, you have not specified where the minimal data set underlying the results described in your manuscript can be found. PLOS defines a study's minimal data set as the underlying data used to reach the conclusions drawn in the manuscript and any additional data required to replicate the reported study findings in their entirety. All PLOS journals require that the minimal data set be made fully available. For more information about our data policy, please see http://journals.plos.org/plosone/s/data-availability
Important: If there are ethical or legal restrictions to sharing your data publicly, please explain these restrictions in detail.

RESPONSE: The City of Nagoya permitted us to analyze the data and publish the results, but we do not have permission to publish the data, even anonymously. The data are managed by the government. We have provided the following contact details:
(Line 127) "Anonymous data were managed by the government of Nagoya."
On website, "Data cannot be shared publicly according to the rule of the local government who provided us the data. Data are available from the Division of Environment Disaster and Health, Environmental Bureau of Nagoya (www.city.nagoya.jp/en/), for researchers who meet the criteria for access to confidential data."

POINT-BY-POINT RESPONSES TO REVIEWS

Reviewer #1: Major comments:
The prevalence of both eczema and wheeze is well put in Table 1. It might be interesting to analyze the group with both eczema and wheeze symptoms (if the data are available from the questionnaire). Infants with both symptoms would have a higher probability of developing asthma than those groups that show eczema alone or wheeze alone. Would this combined prevalence be associated with age, sex/gender, birth order, birth season, and parental allergy history?

RESPONSE: We appreciate your suggestion. We have analyzed the data and added and amended the sentences as follows:
(Line 152) "In infants, the prevalence rates of wheezing were 8%, 17%, and 13%, ~~and~~ those of eczema were 24%, 30%, and 31%, and those of both symptoms were 2%, 7%, and 6% at 3, 18, and 36 months, respectively."
(Line 166) "The tendency in eczema was also observed in both symptoms."
(Line 184) "The significant difference in the first-born children disappeared for both symptoms (Table 4)."

(Line 195) "Parental allergic disease was a general risk factor for ~~both~~ wheezing, ~~and~~ eczema, and both in all age groups."

However, we cannot predict the future asthma prevalence in infants with wheezing and eczema because this is a cross-sectional population survey.
According to the analysis, we have shown the prevalence of both wheezing and eczema in revised Table 1 and added two tables – new Tables 4 and 7.

--In addition to my first comment, I would like to see the prevalence of the combined group since Figure 2 shows some interesting results and differences between the two symptoms, depending on the birth season as well as among the three age groups.

RESPONSE: We have shown the difference in the prevalence of birth seasons in Figure 2.

--On Page 14, "Each correlation with parental allergy history was larger in the older group of infants than in the younger groups." While a monotonically increasing trend is generally observed in Table 2 (Wheeze, 1.45-1.75-2.40, 1.75-2.16-2.79, 1.36-1.50-1.83), Table 3 does not necessarily show this trend across all age groups (1.83-2.33-2.31, 1.70-2.10-2.34, 1.70-2.00-1.83). For instance, comparing 18-month group and 36-month group, it shows a slight decrease in the correlation between eczema and parental allergic disease. Please justify this. Also note that this does not affect the general conclusion of "a strong association with parental allergic diseases in children in the older age group".

RESPONSE: We agree that odds ratios of eczema in the 36-month group were not higher than those in the 18-month group. We have amended the sentences as follows:
(Line 200) "Each correlation on wheezing with a parental allergy history was larger in the older group of infants than in the younger group of infants. In terms of

eczema, the correlation only with maternal atopic dermatitis was larger in the older group."

(Line 280) "and the correlation on wheezing was stronger in older children."

(Line 325) "The data also showed a strong association of wheezing with parental allergic diseases in children in the older age group."

--If the ISAAC survey data is only validated for age 6-7 and 13-14 years, how did the authors scale or normalize the age for younger children? In the last section, the explanation is vague and seems unclear to me.

RESPONSE: We did not validate the modified questionnaire for infants. However, it was already used in other surveys. We have amended the sentences in the Discussion as follows:

(Line 233) "The original ISAAC survey revealed that asthma and atopic dermatitis are more common diseases in the younger age group of schoolchildren"

(Line 241) "respectively, using modified ISAAC questionnaire data"

(Line 313) "We did not have access to any validation study of ~~validated~~ questionnaires for infants. ~~Therefore~~ However, we used the same modified ~~the~~ ISAAC scale for younger children that was also used in ~~as~~ other studies conducted in Japan ~~have also done~~."

--In your conclusion, gender seems to be missing. But Tables 2-5 showed significant differences between male and female participants.

RESPONSE: We appreciate you for pointing out the gender difference. We have changed the sentence in the conclusion as follows:

(Line 323) "The prevalence of both symptoms in males was significantly higher than that in females."

Minor

--et al should be "et al." This should be corrected throughout the manuscript (e.g., Pages 18-20).

RESPONSE: Thank you for notifying this issue. We have corrected the grammatical expression in the text.

In addition, we found the word was used incorrectly. Therefore, we have now revised it – from "multivariate" to "multivariable."

Ref. Hidalgo B, Goodman M. Multivariate or multivariable regression? Am J Public Health. 2013 Jan;103(1):39-40. PMID: 23153131
